# A State-of-the-Art of Functional Scaffolds for 3D Nervous Tissue Regeneration

**DOI:** 10.3389/fbioe.2021.639765

**Published:** 2021-03-18

**Authors:** Maria Grazia Tupone, Michele d’Angelo, Vanessa Castelli, Mariano Catanesi, Elisabetta Benedetti, Annamaria Cimini

**Affiliations:** ^1^Department of Life, Health and Environmental Sciences, University of L’Aquila, L’Aquila, Italy; ^2^Center for Microscopy, University of L’Aquila, L’Aquila, Italy; ^3^Sbarro Institute for Cancer Research and Molecular Medicine and Center for Biotechnology, Temple University, Philadelphia, PA, United States

**Keywords:** brain ECM, neural differentiation, tissue engineering, tissue regeneration, biomaterials

## Abstract

Exploring and developing multifunctional intelligent biomaterials is crucial to improve next-generation therapies in tissue engineering and regenerative medicine. Recent findings show how distinct characteristics of *in situ* microenvironment can be mimicked by using different biomaterials. *In vivo* tissue architecture is characterized by the interconnection between cells and specific components of the extracellular matrix (ECM). Last evidence shows the importance of the structure and composition of the ECM in the development of cellular and molecular techniques, to achieve the best biodegradable and bioactive biomaterial compatible to human physiology. Such biomaterials provide specialized bioactive signals to regulate the surrounding biological habitat, through the progression of wound healing and biomaterial integration. The connection between stem cells and biomaterials stimulate the occurrence of specific modifications in terms of cell properties and fate, influencing then processes such as self-renewal, cell adhesion and differentiation. Recent studies in the field of tissue engineering and regenerative medicine have shown to deal with a broad area of applications, offering the most efficient and suitable strategies to neural repair and regeneration, drawing attention towards the potential use of biomaterials as 3D tools for *in vitro* neurodevelopment of tissue models, both in physiological and pathological conditions. In this direction, there are several tools supporting cell regeneration, which associate cytokines and other soluble factors delivery through the scaffold, and different approaches considering the features of the biomaterials, for an increased functionalization of the scaffold and for a better promotion of neural proliferation and cells-ECM interplay. In fact, 3D scaffolds need to ensure a progressive and regular delivery of cytokines, growth factors, or biomolecules, and moreover they should serve as a guide and support for injured tissues. It is also possible to create scaffolds with different layers, each one possessing different physical and biochemical aspects, able to provide at the same time organization, support and maintenance of the specific cell phenotype and diversified ECM morphogenesis. Our review summarizes the most recent advancements in functional materials, which are crucial to achieve the best performance and at the same time, to overcome the current limitations in tissue engineering and nervous tissue regeneration.

## Introduction

The study of the brain and its development received a considerable attention during the past decades, focusing especially on the analysis of how the processes governing its development can contribute to behavior (Casey et al., 2000, 2005; Makeig et al., 2009; Stevens, 2009; Zehl et al., 2016; Rubin et al., 2017; Yuste and Bargmann, 2017; Wang et al., 2020). The extreme power and complexity of the brain demand the urgent need to increase our knowledge on all significant events occurring during neurodevelopment, in order to improve treatments and therapies for several disorders, such as neural injuries, trauma, stroke, and neurodegenerative diseases. In this context, the scientific community is currently developing new techniques for *in vitro* 3D culture applied to neural tissue. This in particular can be useful also to produce models for *in vivo* architecture, in order to analyze how the brain heals and reacts to trauma and wound (Abud et al., 2017; Tian et al., 2020).

Recent findings show how several typical features of *in situ* microenvironment can be mimicked by using different biomaterials. Previous studies reported about cultivated cells using two-dimensional (2D) plastic consumables, characterized by a solid and robust surface that inhibits the replication of physical, genetic, biochemical environment that occurs during neurodevelopment events (Diekjürgen and Grainger, 2017; Lee et al., 2017). However, such 2D techniques do not allow cells to provide regular and natural reactions, also in terms of morphological aspects, cellular responses, and gene expression modulation (Carletti et al., 2011). Those previous methods helped our current understanding of basic cell biology, however it is now clear that the 2D tissue engineering cannot provide an effective description of the *in vivo* complexity (Birgersdotter et al., 2005).

*In vivo* tissue architecture is characterized by the interconnection between cells and other specific components of the extracellular matrix (ECM). The ECM is a complex 3D network of molecules, regularly rearranged and renovated according to the different events occurring into the tissue. All of its components, including collagen, fibronectin, laminin, and elastin, give support and contribute to mechanical and biochemical signals, depending on their specific cell phenotype (Pu et al., 2018). 3D culture strategy is optimal in this sense, since it allows the recapitulation of all the elements that constitute the ECM, promoting processes such as proliferation, differentiation, migration, and the communication through the activation of unique pathways of the native tissue (Lavon and Benvenisty, 2005). The current research is mostly aimed at optimizing different biomaterials which can be used as scaffolds to sustain neural 3D culture or brain-region specific organoids. In this direction, the use of customized scaffolds could be an efficient way to design novel brain specific organoid prototypes, exhibiting many specific features of the human brain, as they faithfully would reproduce its mechanical, biochemical, and topological characteristics. Thanks to these new biomaterials, scientists will be able to predict and program how progenitor cells attach, proliferate and differentiate *in vitro*, providing more information on the interactions between neurons and glial cells and neural circuits development (Yuan et al., 2018; Yuan and Xing, 2019).

In this review, we collect data and provide a state-of-the-art comprehensive picture of different types of biomaterials for neural repair and regeneration. In particular we focus on the aspects related to the different uses of specific components, as well as the features and the approaches of the construction process. More specifically, we emphasize the interplay between neural cells and different materials, drawing attention towards their potential use as 3D tools for *in vitro* neurodevelopment of tissue models. Until now, scaffolding techniques in neurodevelopment were restricted to neural differentiation control. On the other hand, it seems that the application of engineered biomaterials is able to promote also neural repair and regeneration (Tian et al., 2015; Maclean et al., 2018a), showing the promising findings this pioneering field can lead to. Finally, we focus on how 3D scaffolds can improve the development of new therapeutic approaches for neural tissues, both in physiological and pathological conditions.

## The Interplay Between the ECM and Neural Tissue

**FIGURE 1:**
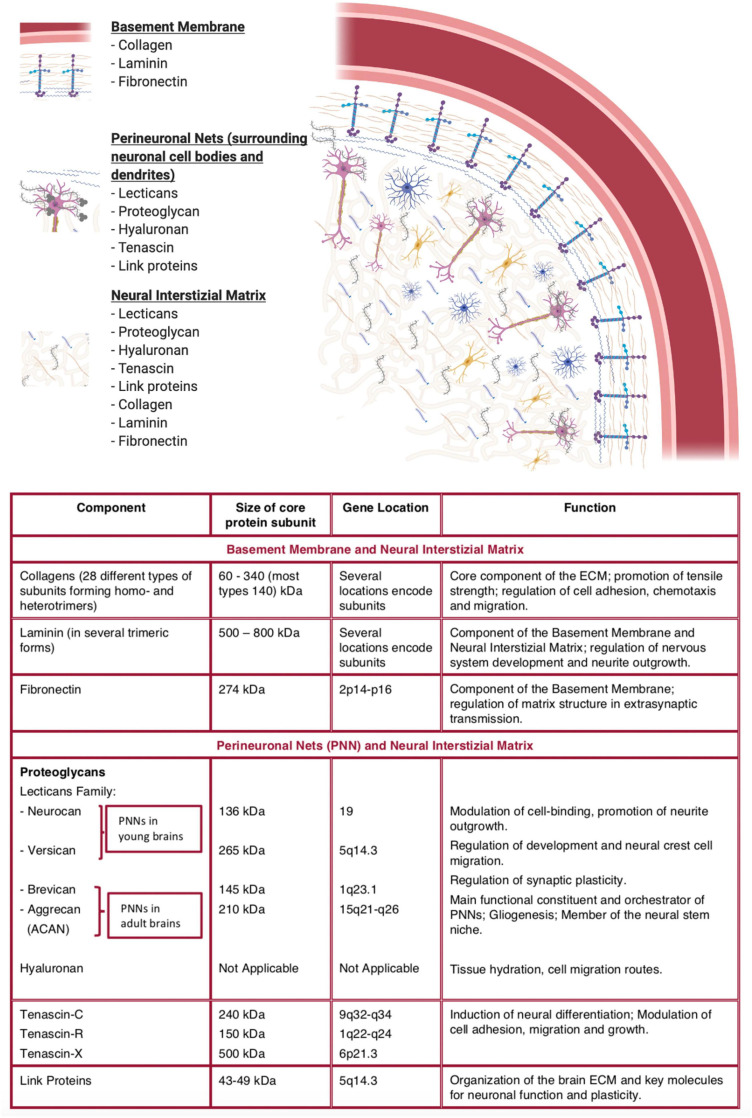
Typical representation of the brain extracellular matrix and description of its key components. (Made with ©BioRender – biorender.com).

The ECM is composed by proteins and polysaccharides situated in the gap among neurons and glial cells, and it represents around the 20% of the full volume of the brain in adults. The brain ECM mostly consists of glycosaminoglycans, proteoglycans, glycoproteins, and low levels of fibronectin, collagen, and vitronectin (Figure 1). Architecturally, the ECM behaves as a natural barrier able to minimize the release of soluble particles and the migration of cells. Furthermore, the ECM regulates various mechanisms during the neural development, and it can perform pathologic and physiologic functions in several processes of adult brain, such as, synapses formation, neurite growth, synaptic stabilization, and injury-related neuroplasticity. For *in vitro* experiments, scientists can use a single purified or several ECM proteins to allow the coating in cell culture. In this way, it is possible to reproduce the complex and heterogeneous molecular structure of the brain ECM to enhance the applications of *in vitro* experiments. Indeed, by isolating the native ECM deriving from the decellularized tissue, it is possible to prepare tissue-specific scaffolds, characterized by the maintenance of the complex cellular and molecular architecture of the ECM. Those specific ECM scaffolds may either be transplanted into animal models, and be used for *in vitro* models, to reproduce the *in vivo* conditions and to investigate the distinct responses on cell behavior. Both rodent and pig brains may be used to obtain decellularized brain tissues. The native ECM of the brain may be isolated to form the coating for 2D applications, or it may be also used as a source of biochemical signals in 3D applications in cell cultures. It has been demonstrated that ECM particles, isolated from brain tissues, stimulated an increased growth of neurites and improved neurons viability (DeQuach et al., 2011; Medberry et al., 2013; Sood et al., 2016). For 3D applications, it has been proved that brain ECM promotes neurons and neural stem cells (NSCs) viability, supports the differentiation of neurons from NSCs, and it improves the growth of axons, assembling a compact structure within the biomaterial (DeQuach et al., 2011; Crapo et al., 2014; De Waele et al., 2015; Sood et al., 2016). Even though the ECM shows biocompatibility with native brain neurons, the decellularized tissue may exhibit incompatibility with neuroglial cells. This occurs in particular after an infection or injury or during inflammatory processes.

The functional characteristics of neuronal and neuroglial cells, such as their electrical activity, may be assessed by using multi-electrode arrays. In this way, it is possible to monitor their *in vitro* connective activity with the structure, in the formation of the 2D model. Multi-electrode arrays are also used to assess the impact of compounds on the activity of neurons in terms of their biochemical and therapeutical functions (Hong and Lieber, 2019). It is essential to investigate and validate the correlations between *in vitro* and *in vivo* systems, to obtain the recapitulation of the morphologic and physiological aspects *in vivo*. In this perspective, through the combination of ECM molecules, specific brain tissues, and neuronal or glial cells, it is possible to create more complex *in vitro* systems, to gain a higher accuracy on neuronal *in vivo* functionality and to supply more significant evidence in terms of response to treatments. Moreover, it is intriguing to evaluate the real feasibility of these applications by studying the association between ECM molecules and the formation and functionality of the neuronal network to develop an *in vitro* model suitable for *in vitro* studies, to gain more potential towards new findings and therapies. It is well established that, the physical interaction between cells and ECM strongly influence cell fate in terms of genetic, chemical, and molecular functions (Zajac and Discher, 2008; Wang et al., 2018; Chighizola et al., 2019; Pereira et al., 2019).

For this reason, the development of scaffolds able to mimic several features of the ECM is crucial to improve the regulation of processes, like the development or stem cell differentiation. Moreover, it is also essential to consider other features related to mechanical properties and biological synthetic scaffolds, such as the biological functionalization and the nanotopography, as such properties increase the regulation of cell behavior and their fate (Xia et al., 2020). Recently, scientists focused their attention on the development of new multidisciplinary techniques to engineer synthetic tissue surrogates and create neural tissue models, in order to provide a better understanding of the mechanisms of brain development, clarifying molecular aspects related to gene expression and its influence on indirect stimuli. The ECM can regulate its activity through the action of several growth factors, behaving as a source of proteins that can be released in an organized manner. This happens for instance in the regulation of stem cell niches (Tang et al., 2020). These growth factors, like epidermal growth factor (EGF) and fibroblast growth factor (FGF), are routinely used for *in vitro* cell culture applications of neural progenitor cells (NPCs; Ochi et al., 2016). Intensive investigations are currently being examined, to reproduce an ECM structure considering the expression of growth factors into the 3D scaffold. In this context, many studies have been directed, taking into consideration the interaction between growth factors and NSCs, adopting specialized scaffolds supplied with immobilized growth factors or small particles able to deliver specific growth factors (Mahoney and Saltzman, 2001). The most relevant families of growth factors for the promotion of tissue repair processes and the development of NSCs towards different lineages, include nerve growth factor (NGF; Xia and Lv, 2018; Li et al., 2020; Ye and Gong, 2020), bone morphogenetic proteins (BMPs; Hart and Karimi-Abdolrezaee, 2020), FGF (Chen et al., 2020), glial cell-line derived neurotrophic factor (GDNF) family ligands (Barker et al., 2020), platelet-derived growth factors (PDGF) family receptors (Sowa et al., 2019), Wnt proteins and Hedgehog (Faniku et al., 2020) proteins. However, the target and the retain of the required concentration of these factors at the site of injury remains still a considerably complicated phenomenon. Current studies aim at understanding how growth factors bound to advanced 3D methods can perform their activity. Bioengineered materials contribute to understand the connections between ECM and NSCs.

## Neural Interaction With Biomaterials for Directing NSCs Fates

**FIGURE 2:**
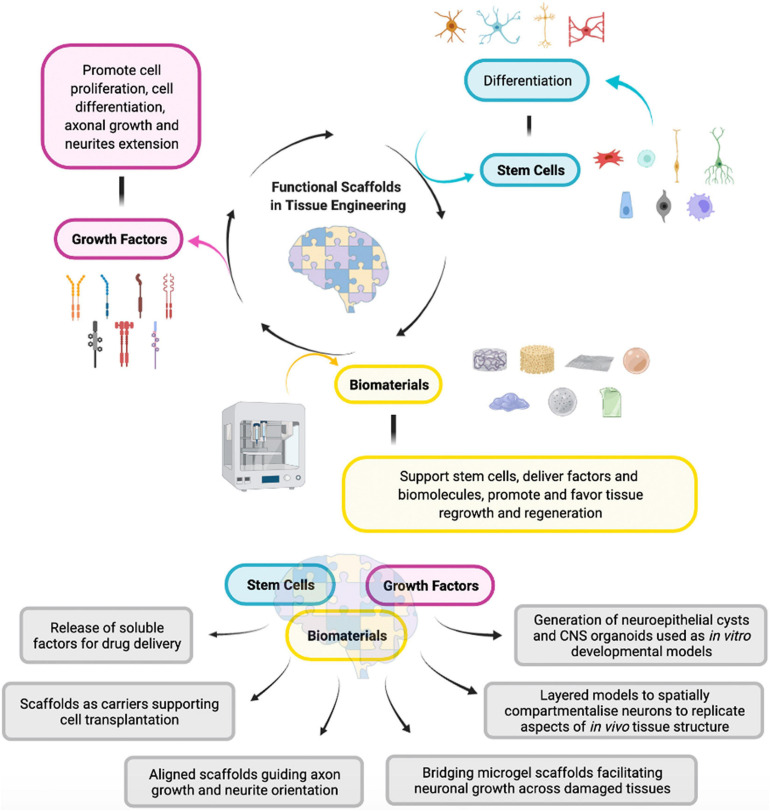
Interplay between the three principal elements involved in the development of a functional scaffold in tissue engineering. (Made with ©BioRender – biorender.com).

In order to have a thorough comprehension of all the different aspects involved in stem cell regulation, in the perspective of increasing their capacity, it is crucial then to study well-characterized scaffolds. Stem cell activity depends on the mechanical and chemical characteristics of a specific substrate, and in addition, it is essential to consider the different source of stem cells used for the specific application together with the kind of tissue and their developmental stage (Figure 2). The interplay between stem cells and substrates of different dimensionality may lead to modifications in the properties and the fate of the cells, in particular concerning self-renewal, adhesion, and differentiation processes (Chen et al., 2013; Eroshenko et al., 2013; d’Angelo et al., 2019). Substrates affect these properties thanks to mechanotransduction, through which cells sense mechanical stimuli as viscosity, elasticity and nanotopography, translating them into physical and chemical signals (d’Angelo et al., 2019). First of all, an appropriate selection of the biomaterial, based on parameters, such as its biological and mechanical compatibility, resistance, physicochemical properties, is essential to determine the suitable application in the field and also the expected stem cell fate for optimal use. It has also been demonstrated that on the same substrate, stem cells can behave differently or even contrarily, depending on their developmental stage. For this reason, it is important to set the correct parameters depending on the biomaterial and the stem cell category, to adopt the appropriate experimental plan, both in terms of feasibility and time efficiency. However, there is not much evidence about the analysis of these guidelines applied to the study of the interaction between biomaterials and stem cells. Consequently, it is useful to perform a systematic study to investigate the biocompatibility of the biomaterial with stem cells, even combining high throughput analysis with combinatorial studies, for example, biomaterial arrays (Teo et al., 2013; Tong et al., 2015), to evaluate the combination of compound characteristics, scaffold parameters such as topography or growth surface, and stem cells features. Although these approaches can be quite expensive, mainly considering the use of several nano-sized topography methods in combo with lithography, they are the most effective procedures to create detailed topographies. For these reasons, it is now necessary to investigate the best procedures in order to achieve the proper combination and the best relationship between biological, mechanical, chemical parameters of the biomaterials, and stem cells properties (Genzer, 2012; Fusco et al., 2015; Alegret et al., 2019). In this direction, for example, it has been demonstrated that recombinant adhesive proteins or human serum can be managed to analyze the specific interplay between a biomaterial and a specific stem cell category, rather than using ECM elements or adhesion proteins from animal derivation for the coating biomaterials (Dzhoyashvili et al., 2015). Moreover, recombinant adhesive proteins have been promoted as a class of ECM mimics, because of their efficiency for coating biomaterials (Wilems et al., 2019), since they are able to regulate the attachment and the expansion of stem cells. There is a growing interest in the development of coating approaches in order to influence stem cell attachment and, subsequently, to maximize the working potential of the stem cell fate. The impact of biological parameters used in these approaches must be considered, for instance in sterilization procedures, cell culture media, and substrate composition. In this regard, the application of specific technologies requires the control of different quality parameters. It is essential to monitor the nature of the biomaterial used for the scaffold preparation, its surface coating, its nanotopography and biocompatibility together with the sterility of the equipment used for its preparation, paying a special attention to internal laboratory quality controls, in order to get the best biomaterials, also in terms of reproducibility and lack of batch-to-batch changes. Together with cell biology parameters, it is also important to consider all the mechanical aspects, such as signaling pathways, the regulation of transcription factors, or gene expression analysis to better understand cell attitude and predict stem cells response in regard of the provided biomaterial. For example, stem cell response depends on the stiffness of the tissue, controlled by the ECM components, through the action of gap junction, soluble factors and surface receptors, and their arrangement. Stem cells are capable to adjust the strength and physical features of their cytoskeleton, producing stress forces that are sent to the surrounding setting (Hoffman et al., 2011). The cell response to mechanical stimuli is known as mechanotransduction, and it also considers the involvement of specific transcription factors. For example, any modification in the organization of the cytoskeleton may affect the localization of mechanosensitive transcription factors, such as yes-associated protein/transcriptional coactivator with PDZbinding motif (YAP/TAZ) or myocardin-related transcription factor-A (MRTF-A). Moreover, RhoGTPase activity impacts on differentiation through the activation of ERK/MAPK pathway (Green and Brown, 2019; Humphries et al., 2019; Kechagia et al., 2019; Sun et al., 2019). The cytoskeleton reorganization can also promote modifications in the nuclear architecture through its relation to Linker of Nucleoskeleton and Cytoskeleton (LINC) complex, able to influence the spatial chromosome organization and gene expression as a result of the mechanical regulation of transcription factors (Uhler and Shivashankar, 2017). In addition, other important features have to be considered, such as the presentation of the ligand and the substrate topography, particularly during neurogenesis, or processes like cytoskeletal rearrangement and the interplay with the extracellular components. Integrins bind the ECM components, mediating the signaling pathways which regulate mechanotransduction and molecular pathways regulating gene expression, development, and differentiation (Wang et al., 2016). Scaffolds substrates can be modified using engineering approaches with nanoscale characteristics to control the NSC’s behavior (Krishna et al., 2016). For example, by using a solvent free nanoscale technique, Poly(ε-capro-lactone) nanowires were able to affect PC-12 cell viability, adhesion and proliferation respect to smooth PCL surfaces. In fact, cells could interact with PCL nanowires through their filopodia and lamellopodia, as shown by SEM imaging and immunofluorescence experiments (Bechara et al., 2010). Considering the differences between 2D and 3D systems, it is fundamental to evaluate the synergy between stem cells and ECM, both in pathological and in non-pathological conditions, to better understand the physiology of the normal neural tissue and to convert the know-how and expertise to improve therapeutic approaches to treat neurological disorders (Cembran et al., 2020). The ECM has a basement membrane characterized by a functional and adaptable architecture that allows the regulation of cell’s response and fate. Moreover, it possesses a typical nanofibrous structure, which reflects the relevance of the substrate topography. For this purpose, scientists have tried several approaches to use electrospun components to simulate the *in vivo* nanofibrous morphology, confirming again that these physical components are responsible for cells fate and response. For example, a network composed of electrospun polyethersulfone (PES) coated with laminin significantly regulates the proliferation and differentiation of NSCs (Wang et al., 2010). The role of specific electrospun nanofibrous scaffolds was examined, therefore demonstrating that thanks to the fiber diameter, it is possible to regulate cell responses efficiently, towards a specific differentiation and proliferation grade, depending on the different conditions. Increased fiber diameter is related to a decrease in terms of proliferation, indicating that the rearrangement of the cytoskeleton regulates and increases cell proliferation. The authors proved that adhesion, migration and cellular differentiation are related to each other, in fact cells seeded on 283 nm fibers were characterized by a glial morphology with random spreads on the fibers mesh, differently from cells seeded on bigger 749 nm fibers, which acquired a neuronal specification. Based on these results, it is clear that the interplay between the scaffold and the cells defines the morphologic and structural features of the cells. Artificial forces can influence the intracellular signaling in terms of differentiation and proliferation rate. Scaffolds signals, for example, send chemical and physically combined stimulations to the nucleus, in order to induce post-translational modifications. In particular, after the cells’ attachment to the scaffold, mechanotransduction signals send impulses to the cytoskeleton allowing the communication with the nucleoskeleton through bridging proteins, culminating in the reallocation of chromosomes before gene transcription regulation (Haque et al., 2006). Lim et al. (2010) demonstrated that the arrangement of poly(ε-caprolactone) nanofibers affect morphologic and proliferative aspects in adult and embryonic neural stem cells (ANSCs), leading to a neural differentiation influenced by the topography and the alignment of substrates fibers. Indeed, the arrangement specifically depends on the fibers size, proving that at 480 nm, it is possible to reach the maximal neural differentiation, culminating in intracellular transduction signals and cell fate specification. These processes lead to gene expression modifications which, in turn, influence components bioavailability and specific cell direction.

Furthermore, scaffolds composed by polyphenylsulfone (PPSu) with distinct topography, characterized by both irregular and aligned electrospun fibers, can influence differently NSC fate (Hajiali et al., 2018). The PPSu scaffold composed by aligned nanofibers increases axonal development and expansion, resulting in an enhanced calcium activity, confirming the creation of higher neural connections in comparison to a regular 2D model. This study showed the capacity of the fiber alignment to influence cell fate, especially for axons, allowing a better comprehension of neural tissue connections. Moreover, it has been demonstrated that scaffolds with electrospun PCL fibers with distinct diameters can create a knot able to imitate physical aspects typical of neural development, such as ca. 1 mm fibers as radial glia and ca. 10 mm as small vessels, resulting in modifications in morphology and in different NSCs fate and responses (Calhoun et al., 2019). In detail, neurospheres seeded on tiny fibers structures show a trend toward migration and extensions processes, whereas neurospheres seeded on bigger fiber structures display a rounded morphology but no morphological modifications with essential communication with the fibers. In this study, it was observed that imitating the native structure of the fibers in the developing brain, the scaffold topography is capable of the regulation and modulation of neuronal precursor cells, occurring through a mechanism that involves the cooperation of integrin, talin, and vinculin. The latter is responsible for the direct transmission of signals from the extracellular context to the cytoskeleton via talin–integrin complex, activated after the induction of strong signals, such as force transmission at focal adhesions (FAs), assuming that topography influences migration and many modifications in morphology (Zhou et al., 2017).

Apart from electrospun scaffolds, other varieties of advanced scaffolds have been developed, in order to minimize their invasiveness for delivery processes in the brain tissue (Eggermont et al., 2020). In a recent study, Béduer et al. (2015) developed a cryogel technique for scaffold creation, to facilitate the development and the extension of neuronal network starting from primary cells. In the seeding and attachment procedure, several physical parameters, as volume, size, and interconnection, need to be considered, in order to increase cell adhesion on cryogel structure. To improve the adhesion and the extension of neurites, also adhesive factors were used in a combo of materials like laminate and poly-L-ornithine. In particular, in this investigation, the neurite extension on the biomaterial walls and in the small gaps inside the architecture of the scaffold was analyzed. Moreover, cells seeded at higher density displayed a well-established and multi-layer network, highlighting the relevance of the interplay between cells and the matrix, to achieve a neuronal tissue as natural as possible in favor of its development (Béduer et al., 2015). Considering the microenvironment features and its ability to guide cell responses and fate, it is essential to highlight the mechanisms that clarify the connection between external stimuli and intracellular modifications and responses. The ability of cells to reorganize their biological and biochemical features of the molecules constituting their enclosing matrices was observed. For example, integrins and other proteins connected to them, contribute to the creation of a functional and peculiar environment with the biomaterial. For these reasons, scientists focused their attention on the design and classification of molecules within the ECM, such as fibronectin, collagen, and laminin, to understand the molecular processes that control neurogenesis, based on their characterization (Long and Huttner, 2019).

Self-assembling peptides (SAPs), are a peculiar class of ionic-complementary peptides, composed by alternate hydrophilic and hydrophobic amino acid residues, which have been used instead of recombinant or animal-derived proteins (Maclean et al., 2016). The peculiar spatial configuration of interactors in the molecules regulates their connections to receptors and influences stem cell fates. It has been demonstrated that laminin-derived sequences YIGSR and IKVAV, and fibronectin-derived sequences RGD and PHSRN, support neurite development, cell attachment, and neuronal differentiation (Sehgal and Banerjee, 2013; Zou et al., 2014; Sun et al., 2016; Marchini et al., 2020). Other important features that contribute to influencing these processes are the presence of particular sequences of peptides and moreover the peptide affinity and spacing. Therefore, stem cell response is directly determined by the density and the affinity between peptides and biomaterial features, as demonstrated by Choi et al. (2015), who showed how the substrate coupling strength of integrin-binding ligands modulates adhesion, spreading and differentiation of human mesenchymal stem cells. In a recent paper, it has been proved that, depending on the compatibility, peptide concentration, and on the stiffness of the scaffold, several events can occur in terms of neurite outgrowth, cell attachment, and development (Yang et al., 2017). Some functional soluble ligands, such as neurotrophins, are essential for their influence in the regulation of neural processes and development. Neurotrophins can trigger two distinct families of Trk and TNF receptors and regulate the expression of proteins responsible for neural survival, cell fate, and responses (Fon et al., 2014; Skaper, 2018). Therefore, the best approach is the design of biomaterials optimized with most fitting parameters compatible with peptides sequences, to increase the functional biological properties of scaffolds (Gaharwar et al., 2020). However, there are still a few difficulties concerning the inability to regulate dimensional and temporal delivery. In this regard, SAPs represent an ideal bio-functional scaffold substrate due to its capacity to preserve and sustain the release of several neurotrophins and other soluble factors, such as BDNF and GDNF (Mohtaram et al., 2013; Rodriguez et al., 2018; Moriarty et al., 2019). In addition, the inclusion of neurotrophins into electrospun scaffolds composed by SAPs allowed a better temporal regulation of the release of several factors (Pugliese et al., 2018). Moreover, it has been demonstrated by Newland et al. (2015) that the use of both star-PEGS cryogel microcarriers and heparin allows the addition of growth factors. These macroporous platforms were applicable to neuronal cultures, providing a good release of growth factors and improving cell survival. They have been also used to better understand the mechanisms through which NSCs failed to regenerate in Alzheimer’s disease (Papadimitriou et al., 2018). To sum up, these new kinds of scaffolds exhibit good capacity into the release of growth factors, essential for neural tissue development and differentiation, and they are also suitable as an important system for many therapeutic approaches in the field of neurodegenerative diseases (Bangde et al., 2017). Thanks to these innovative scaffolds’ features, it will be more and more possible to develop customized and functional biomaterials capable to release neurotrophins, considering the temporal control and delivery, depending on the exact moment when each molecule is essential for the correct control of specific cellular mechanisms.

## Biomaterial Applications for Neural Tissue Engineering

**FIGURE 3:**
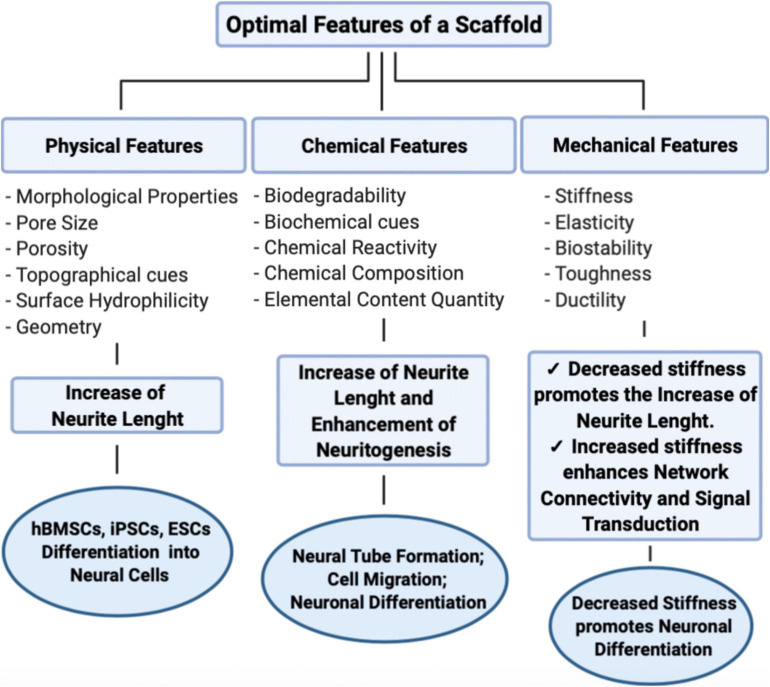
Main properties of an advanced scaffold, and their influence on neural behavior. (Made with ©BioRender – biorender.com).

During the last decades, an important consideration has been given to the classification and application of biomaterials in the field of neural tissue engineering. The ultimate goal is the development of scaffolds reproducing the characteristics of the brain ECM and neurogenic niche to better understand the way neural progenitors, neurons, and stem cells interact with biomaterials. These scaffolds have been developed both for neural progenitors’ support and for axonal extensions. In this context, it is essential to realize a scaffold with excellent features relevant both for *in vitro* and *in vivo* applications, characterized by adjustable physical and dimensional delivery, to translate as more as possible the obtained results for the realization of a specific device optimized for the study of neural development and brain’s cells interactions (Figure 3). For example, through engineering and functionalization processes, it has been possible to develop artificial biomaterials, combining several fields of action, such as biology, chemistry, nanotechnology, and regenerative medicine, to perform 3D cell culture approaches (Shastri, 2006; Zadpoor, 2015; Matai et al., 2020). In the development of tissue engineering techniques, there is a synergistic cooperation of different essential items, namely scaffolds, allowing processes like attachment, growth, migration, differentiation, cells and drugs, or biomolecules helping in cell proliferation and differentiation. In this context, many advanced materials have been employed in the areas of skin repair, muscle, bone and cartilage formation, and neural regeneration. Clearly, one of the most crucial aspects to be considered during skin grafting is the outer environment, which is pivotal for the evaluation of cell’s response, although cells seeded in a single layer provoke atypical responses (Magin et al., 2016; Duval et al., 2017; Gaharwar et al., 2020). In the field of neural regeneration, this aspect is even more relevant, due to the need to incorporate cell approaches and 3D scaffolds to guarantee a proper delivery to enhance the recovery and reorganization of the central nervous system (Schmidt and Leach, 2003; Mahumane et al., 2018; Carvalho et al., 2019). However, there are still some issues related to the recovery and reorganization in the central nervous system, regarding the aspects related to the techniques adopted to favor the cell differentiation process toward neuronal cells. At present, there are several tools promoting cell regeneration, which include the combination of cytokines and other soluble factors delivery through the scaffold, together with different approaches taking into considerations the physical features of the biomaterials, for a better functionalization of the scaffold, improving neural proliferation and cells–ECM interplay. Recently, Maclean et al. (2018b) developed a novel approach, able to regulate the cerebral inflammation process after a trauma. In particular, they developed an efficient strategy for the rearrangement of the cytoskeleton in the brain. They combined the anti-proliferative and anti-inflammatory fucoidan and the SAP approach for *in vitro* and *in vivo* investigations on cell fate after the brain’s trauma (Maclean et al., 2018b). Thanks to this system, it was possible to better understand the molecular key partners occurring during inflammation. In a previous investigation on neural regeneration after stroke, it has been highlighted the importance of innovative biomaterials discovery, using a self-assembling peptide-based scaffold presenting a laminin-derived epitope (IKVAV) in a nanofibrous gel, mimicking so the brain’s major extracellular protein, in order to get a greater comprehension of the specific events which happen during neural regeneration (Somaa et al., 2017). In addition, among all the available biomaterials, hydrogels represent a very interesting class, characterized by an extreme flexibility and a particular ability to modify the biomaterial features in order to get the best achievements in processes such as neural growth, differentiation and regeneration (Madhusudanan et al., 2020). In particular, in terms of physical, chemical and electrical cues, they provide adhesion and support, mechanical stiffness, porosity, and degradability. Moreover, they are also able to arrange a conducive 3D microarchitecture, useful for neural regeneration, starting from their primary basis (Accardo et al., 2018). In addition, Luo et al. (2021) developed a novel treatment to repair large gap peripheral nerve injuries, through the application of bioactive hydrogels combined with dental pulp stem cells.

In terms of biomaterial features, it is important to optimize the biocompatibility and biodegradability of the scaffold, as well as the biological and biochemical characteristics, to create a 3D scaffold able to mimic as much as possible the outer microenvironment to permit cells proliferation and differentiation. For this purpose, innovative biomaterials are emerging in this field to improve the comprehension on trauma and neurodegenerative processes. This especially regards characteristics of the biomaterial architecture, such as nanofiber diameter, arrangement and space size, responsible of neurons adhesion and neurite extension (Lee et al., 2015; Ye et al., 2015). Among the most relevant biomaterials properties, wettability is crucial too, because it is responsible of the fluids’ behavior through surfaces. Indeed, this property depends on the chemical nature of the phases, as surfaces can be hydrophilic or hydrophobic. A hydrophilic surface has a more stable attraction to water, and the degree of the hydrophilic nature depends on the contact angle between the liquid and solid phases. On the other hand, hydrophobic biomaterials are characterized by a poor affinity to water. Regarding the contact angle between the two phases, we can assume that when this is larger than 90°, then it indicates a hydrophobic interaction, whereas an angle smaller than 90° suggests a hydrophilic interaction. Nowadays, hydrophilic and hydrophobic materials can be applied in a significant manner, in many sectors, such as biomedical, anti-fogging techniques for hydrophilic ones; whereas hydrophobic materials can be used to remove petroleum or oil from aqueous solutions, even applied to ceramics and plastics (Drelich et al., 2011; Su et al., 2016; Wang et al., 2017). The goal is the realization of innovative and simplified biomaterials, capable to provide a better understanding of neuronal mechanisms and able to simulate the outer microenvironment of neuronal cells, and at the same time, suitable even for *in vivo* applications (Yang et al., 2020). For these reasons, it is essential to manipulate the mechanical biomaterial properties together with the biological signals to improve proliferation, migration and differentiation. In Figure 3 the most important features and properties of biomaterials used in the field of neural regeneration are provided and compared. Depending on the application, each of these materials is characterized by specific features and different manufacturing techniques.

## Natural Biomaterials for Neural Tissue Engineering

The use of natural biomaterials in the field of neural tissue engineering is extremely advantageous thanks to their biocompatibility and biodegradability properties, together with other specific adaptable chemical characteristics. Usually, natural biomaterials are very similar to substances already present in the organism, and for this reason, they are able to limit the cytotoxicity and immunogenic risks associated with rejection after the implantation in the body. Indeed, natural biomaterials have the ability to be adjustable polymers, and they can be easily managed to accommodate an injury in a delicate and complex physiological geometry. In neural tissue engineering, natural biomaterials can derive from ECM like this happens for collagen, or they can have a marine derivation, like alginate, or from crustaceans, like chitosan. They have been widely used in preclinical studies, working on many different animal models. Table 1 highlights the main natural biomaterials used in neural tissue engineering and their applications.

**TABLE 1:** Natural biomaterials and their application in neural tissue engineering *in vitro* and *in vivo*.

**Natural Biomaterials**				
**Scaffold**	**Method**	**Cell type/model**	**Outcome/application**	**References**
Type I collagen	Hydrogel	Embryonic rat neural stem and progenitor cells	Functional synapse and neuronal network formation in a 3D matrix	Ma et al., 2004
Type-I collagen/hyaluronic matrix	Hydrogel	Embryonic and adult mouse neural stem cells	Survival, proliferation, and differentiation of NSCs and NPCs compared with 2D culture	Brännvall et al., 2007
Agarose	Hydrogel	Adult rats with dorsal over-hemisection spinal cord defect	Functional recovery, tissue repair, delivering neurotrophic factor, stem cell therapy	Jain et al., 2006
Alginate	Hydrogel	Adult rat neural stem cells	First demonstration of the influence of modulus on NSC differentiation in 3D scaffold	Banerjee et al., 2009
	Hydrogel	NIH 3T3 Mouse embryonic fibroblasts	Functional recovery, tissue repair, delivering neurotrophic factors.	des Rieux et al., 2014; Desai et al., 2015
		NIH-3T3 cells (mouse fibroblast-like cells SH-SY5Y cells (human neuronal-like cells Entorhinal cortex slice cultures	Axonal regeneration	Pawar et al., 2015
	Biodegradable Scaffold	Embryonic rat neural stem cells	Functional recovery, tissue repair, stem cell therapy	Hosseini et al., 2016
Cellulose	Hydrogel	Mouse neural stem and progenitor cells	Tissue repair, stem cell therapy, anti-inflammation	Wang Y. et al., 2012
Chitosan	Hydrogel	Embryonic rat neural stem cells	Demonstration of the role of topology in regulating differentiation and proliferation of NSCs in chitosan hydrogels	Wang et al., 2010
	Hydrogel	Rat dorsal root ganglia	Function recovery, axonal regeneration, anti-inflammation, stem cell therapy	Nawrotek et al., 2017
		CA1 region of the adult rat hippocampus	Function recovery, axonal regeneration, delivering neurotrophic factor and drug	Mo et al., 2010
Collagen	Hydrogel	Rat cortical astrocytes	Axonal regeneration, tissue repair, delivering neurotrophic factor, stem cell therapy	Macaya et al., 2013
Gelatin	Hydrogel	Rat adult neural stem cells	Cell survival and proliferation, stem cell therapy	Lim et al., 2012
Hyaluronic acid	Hydrogel	Ventral midbrain-derived mouse neural stem cells	Different mechanical properties influence on the differentiation of NPCs into astrocytes or neurons	Seidlits et al., 2010
	Hydrogel	Subventricular brain-derived adult rat neural stem and progenitor cells	Function recovery, axonal regeneration, tissue repair, anti-inflammation, delivering neurotrophic factor, stem cell therapy	Mothe et al., 2013
		Human induced pluripotent stem cells (iPS)	Cell survival, axonal regeneration, stem cell therapy	Moshayedi et al., 2016
Xyloglucan	Hydrogel	Mouse embryonic cortical neurons and neural stem cells	Axonal regeneration, tissue repair, stem cell therapy	Nisbet et al., 2009

### Collagen

Collagen is the most abundant protein present in the body and is the main component of the connective tissue, providing structure and support. It is also a porous, biocompatible, and absorbable structural biomaterial widely explored in the field of neural tissue engineering. For example, in response to spinal cord injury (SCI), injectable collagen-genipin hydrogel containing FGF-2 has been investigated, confirming its role as a promising candidate for the attraction of astrocytes into the graft of injury (Macaya et al., 2013). Furthermore, it has been observed that neural stem and progenitor cells cultured in 3D collagen gels recapitulate CNS stem cell development, demonstrating active synaptic vesicle recycling and neuronal network formation among collagen-entrapped neurons after injury (Ma et al., 2004). A collagen type I-hyaluronan scaffold has been efficiently used to demonstrate that postnatal neural stem and progenitor cells are able to survive, proliferate and form synapsin I-positive neurons (Brännvall et al., 2007). Moreover, several collagen-based nerve models are commercially ready for use in nerve regeneration. So far, collagen is the only authorized biomaterial for clinical experiments in neural tissue engineering. For example, in a retrospective study, NeuraGen^®^ collagen conduits proved to be safe and effective in 43% of patients with peripheral nerves damage (Wangensteen and Kalliainen, 2010). Another attractive collagen-based nerve guide, called Neuromaix^®^, provided promising perspectives in its first clinical trial, for the bridging of larger nerve gaps in combined nerves (Bozkurt et al., 2017). Indeed, collagen-based nerve models represent the most interesting conduits in clinical settings for nerve regeneration, for their efficacy and biocompatibility, enough to be compared to autologous nerve grafts, the clinical gold standard.

### Gelatin

Gelatin is a natural denatured protein derived from collagen hydrolysis with acid or alkaline. It has been widely used in cosmetics, pharmaceuticals and food products due to its interesting advantages of high biocompatibility, biodegradability, low cost, and availability. In addition, gelatin has a chemical structure which permits to modulate processes like cell adhesion and proliferation, increasing the biological impact of the scaffold after the implantation.

Many studies have confirmed its potential role in association with natural or synthetic biomaterials in neural tissue engineering approaches. Amongst them, the electrospinning approach is particularly interesting because it allows to optimize and manipulate specific mechanical and biological properties of the scaffold, such as nanofiber orientation (Sill and von Recum, 2008). It has been shown that gelatin-hydroxyphenylpropionic acid (Gtn-HPA) hydrogels are promising injectable scaffolds for supporting and influencing adult NSCs, inducing an enhancement in differentiation towards neuronal lineage (Lim et al., 2012). Moreover, gelatin can be used in combination with PCL, acting as a positive regulator of neurite outgrowth and it allows the proliferation of Schwann cells *in vitro* (Gupta et al., 2009). Gelatin can also be combined with PLA, resulting in an increase of motor neuron differentiation and in promotion of neurite outgrowth (Binan et al., 2014). Usually, gelatin is associated with genipin, a safe crosslinker, able to provide stability, bio and cyto-compatibility to the product (Baiguera et al., 2014). Furthermore, gelatin can be used as printable bioink, in fact the use of a combination of gelatin/methacrylamide hydrogel and graphene nanoplatelets has demonstrated a clear architecture, an equal cell distribution and neuronal differentiation (Zhu et al., 2016).

### Hyaluronic Acid

Hyaluronic acid (HA) mucopolysaccharide is one of the major components of connective, epithelial and neural tissues. Thanks to its biological and chemical characteristics it has been widely investigated in the field of tissue engineering. In fact, it is a biodegradable, biocompatible, bioresorbable, and it is able to form hydrogel (Collins and Birkinshaw, 2013). For all these reasons, HA is extensively used in neural tissue engineering, increasing neurite outgrowth, proliferation and differentiation on different substrates. By modifying HA with photocrosslinkable methacrylate groups, hydrogel scaffolds are able to influence on the differentiation of ventral midbrain-derived NPCs into dopaminergic neurons of the substantia nigra (Seidlits et al., 2010). Moreover, the injection of adult brain-derived neural stem/progenitor cells (NSPCs) within a hydrogel blend of hyaluronan and methyl cellulose (HAMC) into a subacute model of rat SCI showed an improved graft survival, an increased oligodendrocytic differentiation, and sparing of perilesional host oligodendrocytes and neurons (Mothe et al., 2013). Furthermore, it has been developed a HA-based self-polymerizing hydrogel to be used as a platform for adhesion of structural motifs and release for growth factors. Thanks to its properties, this optimized HA scaffold promoted survival of human neural progenitor cells (iPS-NPCs) after transplantation into the stroke core and differentially tuned transplanted cell fate through the promotion of glial, neuronal or immature/progenitor states (Moshayedi et al., 2016).

### Alginate

Alginate is a naturally existing anionic polymer obtained from brown seaweed. Due to its biocompatibility, gelation, low cost and toxicity features, it has been widely investigated in tissue engineering. Three-dimensional alginate hydrogel scaffolds have been used to study the proliferation and differentiation of encapsulated NSCs, demonstrating that elastic moduli property of the scaffold influences stem cell fate and increases the expression of the neuronal marker beta-tubulin III (Banerjee et al., 2009). Alginate has been used in several applications, such as growth factors delivery, for example, it has been studied that vascular endothelial growth factor (VEGF)-loaded injectable alginate and fibrinogen hydrogel enhances angiogenesis, neurite growth and plasticity into the lesion site of an injured spinal cord (des Rieux et al., 2014). Due to alginate hydrogels lack of chemoselectivity, they are created using chemical techniques that can be biologically harmful. For this reason, Desai et al. (2015) developed versatile click alginate hydrogels crosslinked via tetrazine-norbornene chemistry, able to increase cell adhesion, maintain structural integrity and favor the delivery of cells and bioactive molecules.

Further, an interesting study showed that anisotropic alginate-based capillary hydrogels (ACH) support peripheral nervous system derived axon growth, together with a migration of slice culture derived astroglia into the hydrogel (Pawar et al., 2015). Also performing *in vivo* experiments, Hosseini et al. (2016) demonstrated that transplantation of NSCs cultured in an alginate scaffold led to a better clinical and histological outcome for recovery from SCI in a rat model. However, one of the limitations of the alginate is the natural presence of impurities, like proteins, endotoxins, heavy metals, or compounds in relation to its marine origin. To overcome this limitation, it is necessary to purify alginate biomaterial through a multi-step extraction technique, to reduce its adverse immunogenic and inflammatory effects in the site of the injection.

### Chitosan

Chitosan is an amino polysaccharide, derived from the chemical deacetylation of chitin obtained from crustaceans and insects. Its chemical properties provide chitosan with a unique set of functional characteristics. Chitosan is extremely versatile, biocompatible, biodegradable, non-cytotoxic, and it presents antitumor and antibacterial activity (Dash et al., 2011). For all these reasons, its great potential has long been recognized. Many evidences highlight the efficient and successful role of chitosan in neural tissue engineering, in particular for its role in cell adhesion and survival, and neurite outgrowth. Chitosan films (Chi-F), chitosan porous scaffolds (Chi-PS), and chitosan multi-microtubule conduits (Chi-MC) were successfully used to investigate their effects on the differentiation and proliferation of NSCs isolated from the cortices of fetal rats. They observed an increase in astrocytes and neuronal differentiation, confirming that topology can have an important role in supporting differentiation and proliferation of NSCs (Wang et al., 2010). Nawrotek et al. (2017) evaluated also the therapeutic effectiveness of a chitosan-based polymer and reported that thermogelling chitosan lactate hydrogels improve functional recovery after a central nervous system damage, by performing *in vivo* experiments. Furthermore, neurotrophin 3(NT-3)-chitosan carriers have been efficiently optimized to evaluate the recovery degree of the cognitive function into an injured brain. Results confirmed the good biological quality of the scaffold material. In particular NT-3-chitosan carriers stimulated the regeneration of axons into the injured brain site and rebuilt the neural circuit, significantly improving the impaired cognitive function (Mo et al., 2010).

### Agarose

Agarose is a natural polysaccharide widely investigated in neural tissue engineering thanks to its biocompatibility, non-toxicity, thermo reversible gelation behavior and physiochemical properties. In addition, agarose can be optimized, by regulating porosity and other mechanical features, to obtain a more efficient axon outgrowth. Agarose scaffolds are able to support 3D neurite extension *in vivo* and it has been demonstrated that they can serve as efficient carriers of drug delivery vehicles. These scaffolds have a great potential thanks to their excellent properties, also in terms of growth increase, neurotrophic factors and anti-scar agents’ delivery and release (Jain et al., 2006).

### Xyloglucan

Xyloglucan is a neutral, non-ionic polysaccharide consisting of a cellulose-like backbone that carries xylose and galactosyl-xylose substituents. In the past decades, xyloglucan-based scaffolds have been developed, to investigate the interaction between neurons and glia within the cultures, in order to establish whether these scaffolds could be used for *in vivo* spinal cord repair. Furthermore, the interplay between xyloglucan scaffolds and NSC cells has been investigated, reporting that functionalized scaffolds were able to support neurons growth, the differentiation of precursors into neurons, and neurite extension under 2D and 3D culture conditions. These data suggest that xyloglucan-based scaffolds can provide a neurotrophic microenvironment (Nisbet et al., 2009).

### Synthetic Biomaterials fOR Neural Tissue Engineering

In order to overcome limitations, such as sourcing, reproducibility, thermal sensitivity, complex chemical structures and processing difficulties that usually demand the use of several solvents, scientists integrate natural biomaterials with synthetic polymers. Synthetic biomaterials used for neural applications in tissue engineering, are produced by using standard chemical reactions, in order to provide a suitable environment and influence a specific stem cell fate. Synthetic polymers can be processed through different techniques, in order to develop scaffolds characterized by different topographies, like nanofibers or microfibers. In addition, they can be used for drug and growth factors delivery applications.

Synthetic polymers can be biodegradable or non-biodegradable. In particular, polyesters of lactic (PLA) and glycolic acid (PGA), and their co-polymer PLGA are considered biodegradable, whereas materials with methacrylate are often non-biodegradable. Originally, scaffolds for neural regeneration were created using the same materials used for surgical repair of peripheral nerves and skin grafts (Madigan et al., 2009). In recent years, thanks to the improvement of biomaterials technology, new models have been developed to increase the quality of materials, creating better tolerated scaffolds specific for the neural microenvironment (Grandhi et al., 2014). Nowadays, neural scaffolds share the same biological and physiochemical properties of the damaged nerve tissue to repair, allowing chemical and architectural adjustments according to the specific needs (Hill et al., 2011; Hopkins et al., 2013; Liu et al., 2017). Thanks to typical features of non-natural materials, such as mechanical strength, flexibility and ease of modification, it is possible to modify structural properties and to use several fabrication methods, like electrospinning, wet-spinning or freeze–drying. However, there are some concerns about the use of synthetic polymers, regarding the presence of several toxic residual monomers coming from partial polymerization, or the presence of degradation products. For these reasons, there is an urgent need in the development of new tests for these polymers, in order to be available and perfectly suitable for the translation to the clinic. Table 2 highlights the main synthetic biomaterials used in neural tissue engineering along with their applications.

**TABLE 2:** Synthetic biomaterials and their application in neural tissue engineering *in vitro* and *in vivo*.

**Synthetic Biomaterials**				
**Scaffold**	**Method**	**Cell Type/Model**	**Outcome/Application**	**References**
Nanofibrous poly (L-lactic acid) (PLLA)	Electrospinning	Immortalized mouse neural stem cell line (C17.2)	Nanofibrous scaffold support NSC differentiation, neurites out-growth, and NSC adhesion	Yang et al., 2004
Mixture of poly (ethylene glycol) (PEG) and poly(L-lysine) (PLL)	Hydrogel	Mouse postnatal isolated neural stem cells	The mechanical modulus of cross-linked hydrogels (PEG/PLL) impacts NSC migration and differentiation	Hynes et al., 2009
Poly(ε-caprolactone) (PCL)	Electrospinning	Mouse cortical NSC/progenitors	Electrospun fibers influence NSC/progenitor proliferation, differentiation, and neurite growth	Wang T.Y. et al., 2012
	Electrospinning	Human Hips Cell-Derived Neuronal Progenitors	Cell survival, stem cell therapy, functional recovery	Havasi et al., 2014
Polyurethane	Hydrogel	Mouse Neural Stem Cells	Cell survival, axonal regeneration, functional recovery, stem cell therapy	Hsieh et al., 2015
IKVAV-RADA16 self-assembling peptide	Hydrogel	Primary mouse neural stem cells	Self-assembling peptide 3D culture for neural tissue applications	Zhang et al., 2010
	Hydrogel	Rat neural stem cells	IKVAV-RADA16 support encapsulated NSCs and reduce the formation of glia astrocytes	Cheng et al., 2013
Fmoc-self-assembling peptides (Fmoc- SAPs)	Hydrogel	Mouse cortical NPCs	SAPs as a tool for cell transplantation	Rodriguez et al., 2014
FGLmx	Hydrogel	Spinal cord-derived neural stem cells	Function recovery, axonal regeneration, stem cell therapy	Wang et al., 2015

### Synthetic Polymers

poly (L-lactic acid) (PLLA), a biodegradable polymer, is a biomaterial whose degradation and mechanical characteristics have been widely investigated. By using an electrospinning technique, scientists developed nanofibrous PLLA scaffolds, characterized by a morphology and architecture similar to those of the natural ECM. So far, these scaffolds were able to mimic the structure and the biological functions of the natural ECM. Moreover, this specific nanostructure, characterized by an increased surface roughness, promoted NSCs adhesion and supported NSCs differentiation and neurite outgrowth (Yang et al., 2004). By using the electrospinning technique, it is possible to recreate the local tissue environment, with a special attention at the definition of fiber alignment, diameter and distance between fibers. In this way, scaffolds can provide physical support for the cells, and at the same time they can maintain the architecture at the damaged site. In particular, it has been demonstrated that fibrous poly ε-caprolactone (PCL) scaffolds, immobilized with glial cell-derived neurotrophic factor (GDNF), are able to promote the survival, proliferation, migration, and neurite growth of transplanted cortical cells, thereby increasing graft integration (Wang T.Y. et al., 2012). Several other studies demonstrated how nanofibers of PCL can enhance growth, proliferation, and migration of various cells. For instance, a significant increase of the adhesion, viability and proliferation of neural progenitors on aligned poly-caprolactone (PCL) nanofibers was reported (Havasi et al., 2014), suggesting a very good compatibility of PCL scaffolds and human iPS cells for neural regeneration. One of the most common polymers used to create synthetic scaffolds in neural tissue engineering is polyethylene glycol (PEG), a biodegradable synthetic polymer of ethylene oxide (EO) units. Thanks to its hydrophilic properties, PEG is highly biocompatible and suitable for its use in hydrogel. It is also biochemically inert and non-immunogenic. Since PEG is not bioactive, it is usually combined with other polymers. For example, Hynes et al. (2009) synthesized a library of 52 hydrogels composed of PEG and poly(L-lysine) (PLL), characterized by independent modifications of chemical and mechanical properties, to investigate the material cues that influence NSC differentiation. By culturing NSCs on these scaffolds, they reported that some combinations of gels were able to promote NSC migration and some other NSC differentiation, suggesting a critical role for elastic moduli in NSC migration and neuronal differentiation (Hynes et al., 2009).

Recently, also polyurethan (PU) has gained attention in the applications for neural regeneration, thanks to its biodegradability and its excellent physical properties. In particular, Hsieh et al. (2015) developed a new 3D bioprinting technique adding NSCs into a thermoresponsive water-based biodegradable PU scaffold. Their results show that in PU hydrogels with the appropriate chemistry and modulus, NSCs had favorable proliferation and neural differentiation, suggesting a potential role for PU hydrogels in the recovery of the function of impaired nervous system in neurodegenerative diseases (Hsieh et al., 2015). Another application in neural tissue engineering considers the generation of synthetic peptide sequences able to assemble into hydrogels. In particular, synthetic peptides are produced through chemical reactions by the action of peptide synthesizers, and they can self-assemble into structures able to support cell growth. It has been demonstrated that specific peptide sequences added to hydrogel scaffolds through self-assembly increase cell adhesion and regulate stem cell behavior. The peptide sequences are mentioned by using a set of one letter abbreviations, one for each amino acid of the sequence. Thanks to their ability to gel upon injection, the use of these kind of scaffolds is very interesting for clinical applications. One of the most frequently used peptides able to self-assembly is RADA, a sequence consisting of 16 repeats of the amino acids RADA. These SAP scaffolds, known commercially under the name Puramatrix^©^, create a β sheet structure, which can bind to other β sheets, developing a novel self-assembling nanofiber scaffold. Zhang’s group evaluated the behavior of NSCs using a IKVAVmx scaffold, created by mixing SAP RADA16 and IKVAV solutions. They found that IKVAVmx scaffold significantly stimulated cell proliferation and migration into the 3D scaffold, and promoted a higher neuronal differentiation, compared to the pure RADA scaffold (Zhang et al., 2010). By performing *in vitro* and *in vivo* experiments, Cheng et al. (2013) confirmed that RADA-IKVAV SAP not only enhanced survival of encapsulated NSCs and reduced the formation of glial astrocytes, but also improved brain tissue regeneration after 6 weeks post-transplantation in a rat brain surgery model. Furthermore, other models of *N*-fluorenylmethyloxycarbonyl self-assembling peptides (Fmoc-SAPs) have emerged as potential biomaterials, thanks to their biocompatibility and their ability to self-assemble through simple interactions into complex nanofibrous hydrogel scaffolds. Fmoc-SAPs delivering cortical NPCs into the mouse brain, showed an improved neural tissue repair through the support of grafted cells and adjacent host parenchyma, and also an attenuation of the inflammatory response for improved tissue repair outcomes (Rodriguez et al., 2014). SAPs got a remarkable interest as a potential approach in neural tissue engineering due to their ability to provide a nanofibrous network structure similar to the native ECM. A class of designed SAP scaffolds, obtained by mixing the RADA sequence with FGL, a motif from neural cell adhesion molecule (NCAM), has been investigated by Wang et al. (2015). They found that non-cytotoxic, biocompatible and bioactive FGLmx scaffolds could promote proliferation of NSCs and induce a differentiation towards the three neural lineages of neurons, astrocytes and oligodendrocytes, suggesting a potential role in SCI regeneration (Wang et al., 2015).

Indeed, the wide collection of synthetic polymers used for neural applications, are characterized by novel topographies, good and unique mechanical and biochemical properties, thanks to which they can now be considered to have a great potential in the field of tissue engineering, although further investigation is needed, to evaluate their effect of *in vivo* treatments and consequent translation to the clinic.

### Conclusion and Future Perspectives

The efficiency of engineered biomaterials is crucial to identify the best approaches in tissue engineering and regeneration. We currently need new techniques and methodologies in order to improve the scaffold performance, depending on their specific use. 3D scaffolds need to ensure a progressive and regular delivery of cytokines, growth factors, or biomolecules, and moreover they should serve as a guide and support for injured tissues. It is also possible to create scaffolds with different layers, each one possessing different physical and biochemical aspects, in order to provide at the same time both organization, support and maintenance of the specific cell phenotype and diversified ECM morphogenesis. Although many efforts have been made in this field, there are still some issues to be solved, in terms of suitability of the scaffold. Scaffolds, including specific biomolecules and growth factors, can present some complications due to the low vitality and resistance. Moreover, the progressive and regular release of factors not always induces the expected effects in the outer microenvironment in terms of functionality, recovery, and efficiency. To these extents, it is to be hoped that 2D and 3D scaffolds optimize their physical, biological, and mechanical features to improve cell adhesion, growth, and differentiation, concerning the specific applications.

To conclude, scientists are trying to focus their studies on the development of advanced and simplified methodologies to produce more efficient and suitable scaffolds containing bio-functional molecules. It is also important to control some issues related to the increased process of neurovascularization, which inhibits necrosis development and graft failing, and even some other secondary side effects which can occur during the process. The solution to those issues would then allow to transfer these approaches to the development of screening tests and *in vivo* procedures, for future clinical applications. In order to achieve this goal, a promising future research perspective lies in studying the mechanical suitability and space restriction in the outer microenvironment, as those characteristics may help cell adhesion, differentiation and regeneration mechanisms.

## Author Contributions

All authors reviewed and evaluated the literature, created the figures, and wrote the article.

## Conflict of Interest

The authors declare that the research was conducted in the absence of any commercial or financial relationships that could be construed as a potential conflict of interest.
